# In Vitro Evaluation of the Effect of Scanning Strategy on the Accuracy of Intraoral Scanners

**DOI:** 10.31661/gmj.v13iSP1.3748

**Published:** 2024-12-30

**Authors:** Mohammad Qadirifard, Mitra Eisaei, Sayed Mohammadreza Hakimaneh, Mohammad Amin Bafandeh, Sayed Shojaedin Shayegh

**Affiliations:** ^1^ Department of Prosthodontics, Faculty of Dentistry, Shahed University, Tehran, Iran

**Keywords:** Accuracy, Precision, Dental Scanners

## Abstract

**Background:**

The accuracy of intraoral scanners depends on the scanning strategy, but evidence on how these strategies affect trueness and precision across scanners is limited. Identifying optimal strategies is key to improving performance and clinical outcomes.

**Materials and Methods:**

A dental cast obtained from an impression of a fully-dentate patient was initially scanned by a laboratory scanner and then by three intraoral scanners namely Trios®4, Carestream 3800, and Medit i700 with three different scanning strategies of A (occlusal surfaces from the left end to the right end, followed by lingual and then buccal surfaces), B (buccal surfaces followed by occlusal and then lingual surfaces from left to right), and C (continuous labiolingual movement with left-to-right direction). Scans were converted to STL format and analyzed in Geomagic for trueness and precision (ISO 5725-1) using ANOVA, Tukey, Welch, and Games-Howell tests (alpha = 0.05).

**Results:**

The effect of scanning strategy was significant on trueness of Carestream (P=0.002) but not Medit and Trios4 (P0.05). In Carestream, the trueness of strategy A was significantly higher than B (P=0.001). The effect of scanning strategy was significant on precision of Medit (P0.001) but not Carestream and Trios4 (P0.05). In Medit, the precision of strategy B was significantly lower than A and C (P0.001 for both).

**Conclusion:**

The scanning strategy’s effect on accuracy varied by scanner type. Strategy A was most accurate in Carestream, while strategy B showed the lowest precision in Medit i700. Other scanners and strategies had similar precision.

## Introduction

A growing trend has been noticed in use of intraoral scanners among dental clinicians in the recent years [1]. Intraoral scanning is initiated by the emission of light on the surface of an object and receipt of the reflected altered light pattern according to the surface geometry of the object by the receptors at the scanner tip [2]. A software then analyzes the light in X, Y, and Z axes to form a mesh. Finally, different scan series are overlapped and stitched to form a three-dimensional (3D) image of the scanned object [3][4]. Following recording of point clouds and stitching of the scans, the scanned object is three-dimensionally reconstructed.

Dental impressions are used to transfer dental information of patients to dental laboratories. However, the impression accuracy is a common concern in this process, since precise recording of details and high dimensional accuracy are the main prerequisites for a successful impression. In order to minimize errors related to impression making and model fabrication, the scanning process should be preferably performed intraorally [5].

Physical impressions obtained by using elastomeric impression materials and custom or prefabricated trays are currently the gold standard dental impressions. Physical plaster casts are subsequently fabricated by pouring the physical impressions. Intraoral scanners directly scan the intraoral anatomical structures, and decrease laboratory and clinical steps such as tray selection, preparation of impression material, and fabrication of plaster model [6]. Furthermore, the digital impression technique is superior to the conventional technique due to elimination of the risk of allergic reactions and no contact between the impression tray and intraoral tissue [7]. Nonetheless, further investigations are still required to cast a definite judgment regarding the possibility of replacement of the conventional method with a fully digital workflow.

For a precise scanning, the object should be positioned at the center of the viewfinder, and the intraoral scanner tip must be moved by the clinician along a specific path, referred to as the scanning strategy [8]. The effects of scanning strategy on the impression accuracy have not been fully elucidated. It appears that using a scanning strategy different from the strategy recommended by the manufacturer would decrease the impression accuracy [9][10]. Anh et al. demonstrated that the accuracy of digital models depended on the initiation point of scanning [11]. Also, Oh et al. suggested avoiding vertical rotation of intraoral scanners to increase the scanning accuracy [12]. It has been reported that depending on the method of data acquisition, the scanning strategy may have different effects on the accuracy of intraoral scanners [13].

All scanning systems generate 3D models by stitching of images at different viewpoints [14]. The scanning strategy has a close association with the image stitching software. Too fast movement of the scanner or abrupt changes in the scanning direction may adversely affect the stitching process [15][16].

The accuracy of scanners is usually defined as the level of agreement between the test results and the acceptable reference values [17]. According to ISO5725-1, the accuracy of measurement methods should be assessed by defining their trueness and precision. Trueness indicates the extent to which the test results agree with the actual or acceptable reference values. Precision indicates the level of reproducibility of the findings. The trueness of intraoral scanners is evaluated by superimposition of the data obtained from the scanner on the data obtained from the reference scanner. Precision of a scanner is assessed by superimposition of the data obtained from repeated scanning of the same object.

The scanning strategy plays an important role in successful scanning in terms of both accuracy and time. The scanning strategies are often exclusive based on the type of intraoral scanners and their technology [18]. The manufacturers of intraoral scanners suggest different strategies but there is no evidence supporting the superiority of a particular strategy over the others [19]. Nonetheless, it has been demonstrated that despite the generally high accuracy of intraoral scanners, some scanning strategies are superior to others in terms of trueness and precision [9][19].

Considering all the above, this study aimed to assess the effect of scanning strategy on the accuracy of intraoral scanners. The null hypothesis of the study was that the scanning strategy would have no significant effect on the accuracy of intraoral scanners.

## Materials and Methods

This in vitro, experimental study was conducted on fully dentate mandibular dental arch of a candidate. The study protocol was approved by the ethics committee of the university (IR.SHAHED.REC.1402.105).

### Sample size

The sample size was calculated to be 13 for each group using ANOVA feature (main effect and interactions) of G-Power software assuming alpha=0.05, study power of 0.8, and effect size of 0.4. Since 9 groups (3 scanners and 3 scanning strategies) were assessed in this study, a total of 117 scans were performed. By inclusion of 10 reference scans (5 scans repeated twice by a laboratory scanner), a total of 127 scans were performed.

### Scanning process

**Figure-1:**
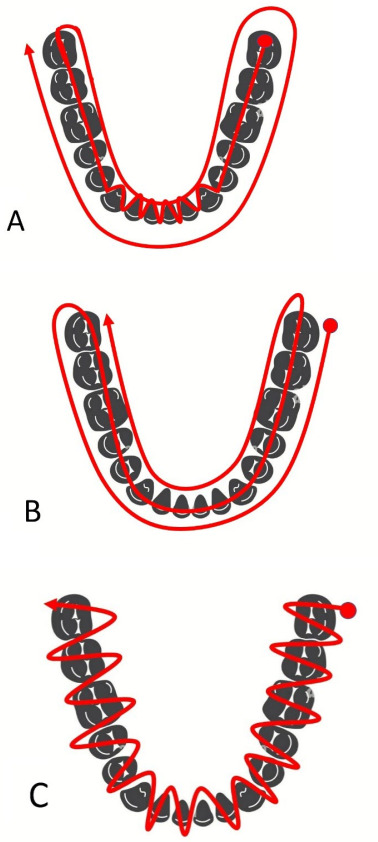
Figure 1. Scanning strategies (A) occlusal surfaces from the left end to the right end, followed by lingual and then buccal surfaces, (B) buccal surfaces followed by occlusal and then lingual surfaces, and (C) scanning was performed with a continuous labiolingual movement with left-to-right direction

After obtaining written informed consent from the candidate, a standard impression was made from his mandibular arch and poured to obtain a dental cast. The cast was scanned by a laboratory scanner (Freedom UHD; DOF Inc., Seoul, South Korea) to serve as the reference scan, and three intraoral scanners namely Trios®4 (3Shape HQ, Copenhagen, Denmark), Carestream 3800 (Carestream Dental LLC, Atlanta, USA), and Medit i700 (Medit Co., Seoul, South Korea) with three different scanning strategies as follows:

Strategy A: Sequential scanning was started from the occlusal surface of the lower left posterior teeth and continued to the anterior teeth with labiolingual movements, and then the occlusal surface of lower right posterior teeth. It was then accomplished by scanning of the lingual and then labial surfaces (Figure-1A).

Strategy B: Sequential scanning was started from the buccal surface with a left-to-right direction, and continued to the occlusal surfaces, followed by the lingual surfaces (Figure-1B).

Strategy C: Scanning was performed with a continuous labiolingual movement with left-to-right direction (Figure-1C).

**Figure-2:**
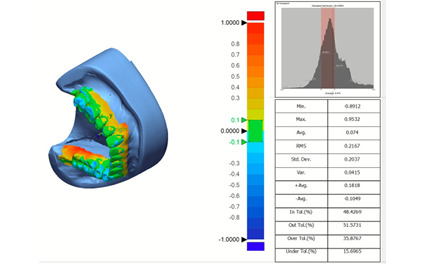
Figure 2. A representative output of the best fit 3D alignment

All scans were performed by the same operator, and 117 meshes were obtained. Also, 5 scans were obtained by the laboratory scanner twice to validate its optimal precision. Trueness, precision, and accuracy were defined according to ISO5725-1 standard. Also, the exclusive software of each scanner was used for intraoral scans, as instructed by the manufacturers. Accordingly, the high-resolution feature was disabled. Also, the scanners were calibrated prior to the procedure in each session. After each scan, the scanning time was recorded, and the files were assessed by the software. All scans were converted to STL file, and imported to Geomagic Control X reverse engineering software version 2022 1.0.70 (3D Systems, Inc., Rock Hill, SC, USA) for further analyses (Figure-2). All STL files were compared with the reference STL file using the best fit 3D alignment. Accordingly, mathematical algorithms with the best fit 3D alignment of the STL file on the reference file were used, and the variance between the files was calculated by their superimposition. The root mean square (RMS) values were used for the comparison of the STL scan file and STL reference file. Lower RMS value would mean higher trueness and precision.

### Statistical Analysis

The normality of data distribution was analyzed by the Shapiro-Wilk test while the homogeneity of the variances was analyzed by the Levene’s test. Accordingly, two-way ANOVA was applied to analyze the effect of scanner type and scanning strategy on trueness and precision. Since the interaction effect of scanner type and scanning strategy was significant, and the homogeneity of the variances was met, trueness was compared by one-way ANOVA among the three groups while the Tukey’s test was applied for pairwise comparisons of the groups regarding trueness. For assessment of precision (due to non-homogeneity of the variances), the Welch test was used to compare the three groups while the Games-Howell test was applied for pairwise comparisons. All statistical analyses were carried out using SPSS version 26 (SPSS Inc., IL, USA) at 0.05 level of significance.

## Results

**Table:** Table1. Measures of central dispersion for trueness based on the scanning strategy and scanner type (n=13)

**Scanner type**	**Scanning strategy**	**Mean**	**Std. deviation**	**Std. error**
Carestream	A B C	0.129 0.146 0.138	0.012 0.009 0.010	0.003 0.003 0.003
Medit i700	A B C	0.159 0.152 0.146	0.014 0.019 0.009	0.004 0.005 0.003
TRIOS^®^4	A B C	0.164 0.158 0.159	0.017 0.016 0.023	0.005 0.004 0.006

**Table:** Table2. Pairwise comparisons of scanners regarding trueness based on the scanning strategy

**Strategy**	**Scanner 1**	**Scanner 2**	**Mean difference** (1-2)	**Std. error**	**P-value**
	Carestream	Medit i700	**-0.029**	**0.006**	**<0.001**
A	Carestream	TRIOS^®^4	**-0.034**	**0.006**	**<0.001**
	Medit i700	TRIOS^®^4	**-0.005**	**0.006**	**0.622**
	Carestream	Medit i700	**-0.009**	**0.006**	**0.34**
C	Carestream	TRIOS^®^4	**-0.022**	**0.006**	**0.003**
	Medit i700	TRIOS^®^4	**-0.013**	**0.006**	**0.09**

**Table:** Table3. Measures of central dispersion for precision based on the scanning strategy and scanner type (n=12)

Scanner type	Scanning strategy	Mean	Std. deviation	Std. error
Carestream	A B C	0.0569 0.0566 0.0566	0.006 0.017 0.013	0.002 0.005 0.004
Medit i700	A B C	0.057 0.1039 0.0607	0.008 0.029 0.016	0.002 0.008 0.005
TRIOS^®^4	A B C	0.0577 0.0622 0.0620	0.015 0.016 0.013	0.004 0.004 0.004

**Table:** Table4. Pairwise comparisons of the precision of scanners in strategy B using the Games-Howell test

**Scanner 1**	**Scanner 2**	**Mean difference** **(1-2)**	**Std. error**	**P-value**
Carestream Carestream Medit i700	Medit i700 TRIOS^®^ 4 TRIOS^®^ 4	-0.047 -0.006 0.042	0.009 0.007 0.009	<0.001 0.677 0.001

**Figure-3:**
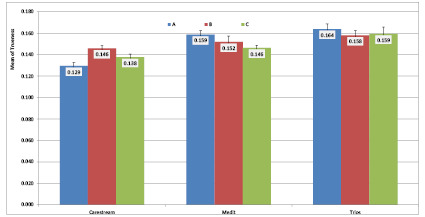
Figure 3. Bar chart of the mean trueness of the three scanners with different scanning strategies

**Figure-4:**
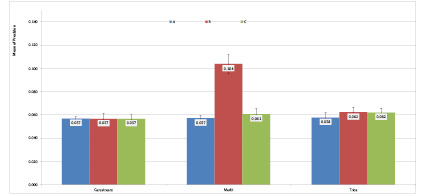
Figure 4. Bar chart of the mean precision of the three scanners with different scanning strategies

A total of 13 scans for each scanning strategy of each scanner and a total of 117 scans were assessed for trueness. In each scanner group, 12 scans for each strategy, and a total of 108 scans were assessed for precision.

### Trueness

Table-1 presents the measures of central dispersion for trueness based on the scanning strategy and scanner type. Two-way ANOVA (due to normal distribution of data and homogeneity of the variances) indicated the significant effect of scanner type (P=0.01) and the interaction effect of scanner type and scanning strategy (P=0.025) on trueness. However, the effect of scanning strategy on trueness was not significant (P=0.468). Considering the presence of a significant difference in trueness among different scanners, pairwise comparisons were performed by the Tukey’s test, which revealed significant differences between all pairs, such that Carestream had a significantly higher trueness than Medit (P<0.001) and Trios (P<0.001), and Medit had a significantly higher trueness than Trios (P=0.05). Considering the significant interaction effect of scanner type and scanning strategy on trueness, one-way ANOVA was applied to assess the effect of scanning strategy on trueness of each scanner group, which revealed that the effect of scanning strategy was significant on trueness of Carestream (P=0.002) but not Medit (P=0.113) and Trios (P=0.704). Considering the significant effect of scanning strategy on trueness of Carestream scanner, the Tukey’s test was applied for pairwise comparisons of scanning strategies. The results revealed that the trueness of Carestream in strategy A was significantly different from strategy B (P=0.001), and the strategy A had a lower RMS (higher trueness) than strategy B. However, the differences between A and C (P=0.136), and B and C (P=0.139) were not significant.

In assessment of the interaction effect of scanner type and scanning strategy on trueness, different scanners were compared for each scanning strategy, which revealed significant differences in strategies A (P<0.001) and C (P=0.004), but not B (P=0.153). Pairwise comparisons (Table-2) revealed significant differences in trueness between Carestream and Medit (P<0.001), and also Carestream and Trios (P=0.001) for strategy A, and between Carestream and Trios (P=0.003) in strategy C. No other significant differences were found (P>0.05). Figure-3 presents the bar chart of the mean trueness of the three scanners with different scanning strategies.

### Precision

Table-3 presents the measures of central dispersion for precision based on the scanning strategy and scanner type. Normal distribution of data was confirmed, but the assumption of homogeneity of the variances was not met (P=0.001). The results showed significant effects of scanner type, scanning strategy, and their interaction on precision (P<0.001 for all). Accordingly, the Welch test was applied to assess the effect of scanning strategy based on the scanner type on precision. The results showed that scanning strategy had no significant effect on precision of Carestream (P=0.997) and Trios (P=0.713), but had a significant effect on precision of Medit (P<0.001). Pairwise comparisons of the scanning strategies for precision by the Games-Howell test showed that strategy A had a significantly higher precision than strategy B (P<0.001), and strategy B had a significantly lower precision than strategy C (P=0.001). The difference between A and C was not significant (P=0.774). The Welch test showed no significant difference in precision of different scanners in using strategy A (P=0.177) and strategy C (P=0.928). However, the difference in this regard was significant in strategy B (P=0.014). Pairwise comparisons of the scanners in using strategy B by the Games-Howell test (Table-4) revealed significantly higher precision of Carestream than Medit (P<0.001), and Trios than Medit (P=0.001). Figure-4 presents the bar chart of the mean precision of the three scanners with different scanning strategies.

## Discussion

This study assessed the effect of scanning strategy on the accuracy of intraoral scanners. The null hypothesis of the study was that the scanning strategy would have no significant effect on the accuracy of intraoral scanners. The results showed that the trueness of Carestream scanner was significantly higher than that of Medit and Trios in all strategies. In other words, it had a higher accuracy than other scanners. Also, the difference in strategies A and C was significant in Carestream but not in other scanners. Carestream had higher trueness (lower RMS value) compared with the other two scanners in using strategy A, and compared with Trios in strategy C. The Carestream intraoral scanner uses the Active Speed 3D Video technology which records the images in full HD quality, resulting in better reading of the finish line and higher scanning accuracy. Assessment of precision revealed that scanning strategy had a significant effect only on precision of Medit, such that strategy A yielded the most accurate, and strategy B yielded the least accurate results regarding precision. Also, the difference in precision of scanners was only significant in strategy B with significant differences between Carestream and Medit, and also Medit and Trios. Thus, the null hypothesis of the study was rejected.

In the present study, the RMS value of trueness of scanners ranged from 129 to 164 µm, and Carestream showed the lowest value (higher trueness). Previous studies reported acceptable restoration misfit in the range of 50-120 µm [20], which is lower than the value obtained in the present study. Also, it has been reported that restorations with a misfit > 200 µm would not be acceptable. In digital techniques, the minimum required trueness and precision would be 50 µm and 10 µm, respectively [20]. The precision of scanners in the present study ranged from 56 to 62 µm. Considering the aforementioned values, the trueness and precision of all scans were acceptable in the present study.

It has been reported that error rate is usually higher in S-shaped scanning strategies. Also, the difference between the master model and digital scanner is always increasing in use of patterns with linear movements. Furthermore, the accuracy of digital models also depends on the scanning initiation point [11]. Since a direction change can adversely affect the image stitching process, vertical and rotational movements of the intraoral scanner tip should be avoided [12]. The scanning strategy may differently affect the accuracy of scanners [13][21]. Abrupt changes in the path of scanner also adversely affect the stitching process [15][16]. Also, no time limitation should be set for scanning in the clinical setting, and the manufacturer’s instructions should be precisely followed by an expert operator to obtain favorable results [22].

Furthermore, in reconstruction of 3D models, a higher frequency of errors is often seen in curved areas of dental arch as in the site of premolars and canine teeth and distal surface of molars, which require further angulation of scanner during scanning. Nonetheless, Müller et al. [10], Ender et al. [23], and Wagner et al. [24]. reported that scanning strategy had no significant effect on the accuracy of digital impressions, which was in contrast to the present findings, probably due to assessing different types of scanners and different scanning strategies.

Variations in accuracy of intraoral scanners can be due to a number of reasons such as different physical resolution of scanners [25], trueness of scanning strategy (which cannot be assessed in the clinical setting due to the lack of reference), and combing trueness and precision data, which can improve the performance of scanners.

Rotation of scanner tip enhances scanning in the interproximal areas in prepared teeth and curvatures of anterior teeth [10][26]. According to Medina-Sotomayor et al. [21], zigzag movements have a higher accuracy than straight movements in scanning. Oh et al. [12]. showed that abrupt rotation of scanner tip would impair stitching and decrease the scanning accuracy. Maintaining a distance between the scanner tip and the target tooth during rotation of scanner tip is another possible reason for inaccuracy due to errors in image overlapping process [26]. The hardware and software systems of intraoral scanners can also affect their performance and accuracy [27]. Intraoral conditions and experience and expertise of the operator can also affect the results.

In active triangulation technology, as in Medit i700, the distance of the object from the image axes is calculated at two different visible points 15. Scanners with active triangulation technology are more affected by the type of substrate than scanners operating based on confocal microscopy [28]. Medina-Sotomayor et al. [21]. assessed the effect of scanning strategy on the accuracy of 4 intraoral scanners: two with confocal microscopy, one with active wavefront sampling, and one with active triangulation technology. They demonstrated that only the accuracy of scanner with confocal microscopy technology depended on the scanning strategy. Also, higher accuracy was obtained in using continuous strategy (as in strategy C of the present study). The current study also showed significant effect of scanning strategy on the accuracy of Carestream scanner with Active Speed 3D Video technology but the effect of scanning strategy on the accuracy of Medit i700 (with active triangulation and 3D video technology) and Trios4 (with confocal technology) was not significant. Gavounelis et al. [29]. demonstrated that the accuracy of an intraoral scanner with active triangulation technology was affected by the scanning strategy, and continuous scanning resulted in lower accuracy. They scanned the full dental arch in their study similar to the type of scan required for orthodontic patients. Such differences in the results can be due to using different scanner types and their different software and hardware systems. All systems use the image stitching algorithm for reconstruction of 3D models, which is prone to some errors.

In the present study, the best results in terms of trueness were obtained by using Carestream with strategy A (129 µm), followed by strategy B (149 µm); while, the worst results were obtained by Trios in strategy A (164 µm). Although the obtained values were slightly higher than the range of 50-120 µm, they were all < 200 µm, and were therefore acceptable. Giuliodori et al. [30]. assessed the accuracy of intraoral scanners with different scanning strategies and reported the most favorable trueness and precision for Medit i700. In the present study, Medit i700 yielded results in between those of Carestream and Trios. Kim et al. [31]. compared 10 intraoral scanners and reported the highest trueness in Trios®3.

Scanning strategy may be more important in patients with crowding, and those using orthodontic appliances such as brackets with deep undercuts and translucent or reflective surfaces since these factors may decrease the accuracy of scanning [32][33]. Also, the accuracy of scanners may be related to mucosal topography and presence of structures such as palatal rugae in edentulous patients, and thus, scanning strategy may be an important factor in such patients [34].

It has been reported that vertical movements and rotations of scanner tip should be minimized since they would impair image stitching [12]. Gavounelis et al. [29]. reported low accuracy in strategy C with higher frequency of rotations. In the present study, lower accuracy was noted in strategy B in which, the scanners were mostly held horizontally. The same results were reported by Passos et al. [13], who showed that sequential strategy yielded poorer results than linear strategy. Gavounelis et al. [29]. showed that Medit i500 had < 50 µm error in all scanning strategies. The same results were reported by some others [18][35]. The 50-µm accuracy is also acceptable for 3D printing in the clinical setting. In the digital workflow, single-unit fixed restorations may be fabricated with up to 120 µm marginal misfit [36], although the values obtained in the present study were slightly higher than 120 µm.

This study had some limitations. Many intraoral (temperature, light, moisture), operator-related (scanning pattern, experience and expertise), scanner-related (light source, receptor, capture box), and area-related (scanning area, length, and surface irregularities) factors may affect the accuracy of intraoral scanners [37][38], which were not assessed in the present study, and only the effects of scanner type and scanning strategy were evaluated. Also, blood, saliva, space shortage, mobility of the mucosa, and jaw movements in the clinical setting can affect the accuracy of scanning, which could not be simulated in vitro. Further investigations are required comparing different intraoral scanners based on different image acquisition algorithms to assess the interaction effect of scanning strategy and scanning technology on the accuracy of scanning. Also, the software of scanners, image-stitching algorithms, and guided scanning strategies that would improve accuracy should be further investigated in future studies.

## Conclusion

The effect of scanning strategy on the accuracy of intraoral scanners depended on the scanner type. Strategy A yielded the most accurate scans in terms of trueness in Carestream. The lowest precision was noted in strategy B when using Medit i700. Other scanners and strategies were almost comparable regarding precision.

## Conflict of Interest

The authors have declared that no conflict of interest exists.
